# Risk assessment tools for sexual assault: a scoping review

**DOI:** 10.1192/j.eurpsy.2025.2162

**Published:** 2025-08-26

**Authors:** E. Doody, D. Flynn, S. Nolan

**Affiliations:** 1 Psychiatry, University Hospital Waterford, Waterford; 2 Psychiatry, RCSI, Dublin, Ireland

## Abstract

**Introduction:**

Risk assessment tools are used to enhance patient safety and promote quality care by limiting adverse outcomes to the greatest extent possible. In the Republic of Ireland, risk is monitored and regulated by the Mental Health Commission (MHC). A 2023 report by the MHC identified high numbers of recorded episodes of sexual assault on the acute inpatient unit in the Department of Psychiatry (DOP), University Hospital Waterford (UHW). Standard risk assessment tools are used in the DOP, however, these risk assessment tools failed to identify those who were at high risk of perpetrating a sexual assault.

**Objectives:**

The purpose of this research was to determine if there were risk assessment tools with a higher predictive value of identifying risk of sexual assault in an acute adult inpatient psychiatric setting. I also wanted to establish whether there were risk assessment tools available which assess risk of sexual assault in those with no history of perpetrated sexual assault.

**Methods:**

This scoping review was prepared according to the PRISMA-Scr guidelines. Databases including Embase, Medline, CINAHL, UptoDate, TRIP, Cochrane and PsychINFO were searched. Keywords included inpatient, psychiatry, mental health, risk assessment tools, risk assessment scales, risk management, sexual assault and sexual offense. There was no limit on the date of publication or country of origin of articles. Only articles that used risk assessment tools on adults, who were inpatients in an acute psychiatric setting, in the English language were included. Only risk assessment tools that included assessment of risk of sexual assault were included.

**Results:**

A total of 15 articles were identified. There was a dearth of literate that compared risk assessment tools in this population with regards to risk of sexual assault. Most articles exploring risk of sexual assault focused on the study of those who were offenders and explored the risk of recidivism through the use of risk assessment tools.

**Conclusions:**

Risk cannot be accurately predicted or eliminated. However, determining the most appropriate and comprehensive risk assessment tools, with the highest probability of identifying risk of sexual assault has the potential to enhance patient safety and improve the quality of care provided. There is a lack of risk assessment tools that assess sexual assault, especially in those with no prior history of perpetrating a sexual assault.

**Disclosure of Interest:**

None Declared

